# Is association of preterm birth with cognitive-neurophysiological impairments and ADHD symptoms consistent with a causal inference or due to familial confounds?

**DOI:** 10.1017/S0033291719001211

**Published:** 2020-06

**Authors:** Sarah-Naomi James, Anna-Sophie Rommel, Fruhling Rijsdijk, Giorgia Michelini, Gráinne McLoughlin, Daniel Brandeis, Tobias Banaschewski, Philip Asherson, Jonna Kuntsi

**Affiliations:** 1 King's College London, Social, Genetic and Developmental Psychiatry Centre, Institute of Psychiatry, Psychology and Neuroscience, De Crespigny Park, London, UK; 2 MRC Lifelong Health and Ageing Unit at UCL, University College London, London, UK; 3 Department of Child and Adolescent Psychiatry and Psychotherapy, Central Institute of Mental Health, Medical Faculty Mannheim/Heidelberg University, Mannheim, Germany; 4 Department of Child and Adolescent Psychiatry and Psychotherapy, Psychiatric Hospital, University of Zurich, Zurich, Switzerland; 5 Center for Integrative Human Physiology, University of Zurich, Zurich, Switzerland; 6 Neuroscience Center Zurich, University of Zurich, Zurich, Switzerland

**Keywords:** Adolescents, causal pathways, cognitive impairments, ERP, preterm birth, sibling-comparison

## Abstract

**Background:**

Preterm birth is associated with an increased risk for cognitive-neurophysiological impairments and attention-deficit/hyperactivity disorder (ADHD). Whether the associations are due to the preterm birth insult *per se*, or due to other risk factors that characterise families with preterm-born children, is largely unknown.

**Methods:**

We employed a within-sibling comparison design, using cognitive-performance and event-related potential (ERP) measures from 104 preterm-born adolescents and 104 of their term-born siblings. Analyses focused on ADHD symptoms and cognitive and ERP measures from a cued continuous performance test, an arrow flanker task and a reaction time task.

**Results:**

Within-sibling analyses showed that preterm birth was significantly associated with increased ADHD symptoms (*β* = 0.32, *p* = 0.01, 95% CI 0.05 to 0.58) and specific cognitive-ERP impairments, such as IQ (*β* = −0.20, *p* = 0.02, 95% CI −0.40 to −0.01), preparation-vigilance measures and measures of error processing (ranging from *β* = 0.71, −0.35). There was a negligible within-sibling association between preterm birth with executive control measures of inhibition (NoGo-P3, *β* = −0.07, *p* = 0.45, 95% CI −0.33 to 0.15) or verbal working memory (digit span backward, *β* = −0.05, *p* = 0.63, 95% CI −0.30 to 0.18).

**Conclusions:**

Our results suggest that the relationship between preterm birth with ADHD symptoms and specific cognitive-neurophysiological impairments (IQ, preparation-vigilance and error processing) is independent of family-level risk and consistent with a causal inference. In contrast, our results suggest that previously observed associations between preterm birth with executive control processes of inhibition and working memory are instead linked to background characteristics of families with a preterm-born child rather than preterm birth insult *per se*. These findings suggest that interventions need to target both preterm-birth specific and family-level risk factors.

## Introduction

Preterm birth occurs in 8.6% of births in developed countries (Blencowe *et al*., 2012), and has many known risk factors such as low socio-economic status, low maternal educational status, maternal pre-existing health problems and maternal genetic risk (Goldenberg *et al*., 1996, 2008; Plunkett and Muglia, 2008; Blencowe *et al*., 2012). Whilst survival rates of preterm-born children are improving, many negative long-term outcomes have been implicated, including academic difficulties (Moster *et al*., 2008), cognitive-neurophysiological impairments (Potgieter *et al*., 2003; Johnson *et al*., 2011; Lee *et al*., 2011; Rommel *et al*., 2017) and an increased risk for neurodevelopmental disorders (D'Onofrio *et al*., 2013), in particular attention-deficit/hyperactivity disorder (ADHD) (Bhutta *et al*., 2002).

Whether the association between preterm birth and the negative outcomes is due to the preterm birth insult *per se*, or due to other environmental or genetic risk factors that characterise families with preterm-born children (including individuals born at term), is difficult to disentangle as preterm-born children have often been compared to unrelated controls who may have differed on unmeasured risk factors (Thapar and Rutter, 2009). While twin study designs are not well suited to address this issue given that twins are typically exposed to the same birth event, using non-twin siblings in a sibling-comparison design allows the comparison of preterm birth outcomes whilst controlling for all familial risk factors shared by siblings, such as socio-economic status and maternal genetic risk (Donovan and Susser, 2011; D'Onofrio *et al*., 2013, 2014; Skoglund *et al*., 2014). If there are within-sibling associations between preterm birth and specific outcomes, which controls for unmeasured familial confounding factors, this is consistent with a causal effect of preterm birth. Using this approach, a large Swedish epidemiological study found a dose-response relationship between earlier gestational age (GA) and increased likelihood of an ADHD diagnosis, independent of familial confounds, in line with a causal inference (D'Onofrio *et al*., 2013). However, no study, to our knowledge, has applied the sibling-comparison approach to investigate cognitive and neurophysiological impairments associated with preterm birth.

We recently performed detailed investigations of the cognitive-neurophysiological impairments in preterm-born adolescents, when compared to unrelated term-born control adolescents, and, in order to help understand the increased risk of ADHD in preterm-born individuals, to term-born adolescents with ADHD (James *et al*., 2017; Rommel *et al*., 2017, 2019). Compared to the unrelated control group, the preterm group showed impairments that were similar to those observed in the ADHD group in decreased working memory, IQ, cognitive measures of preparation-vigilance [mean reaction time (MRT) and reaction time variability (RTV)], event-related potential (ERP) activity of response preparation, response inhibition, conflict monitoring and error processing (James *et al*., 2017; Rommel *et al*., 2017, 2019). The preterm group was further uniquely impaired on ERP activity of executive response control, compared to both ADHD and control groups (Rommel *et al*., 2017). In addition, in a simple reaction time task, the introduction of rewards and a faster event rate produced greater improvements from baseline (slow, unrewarded) conditions in both ADHD and control groups in ERP measures of attention allocation and response preparation, but not in the preterm group (James *et al*., 2017). Overall, preterm-born adolescents showed both ADHD-like and unique impairments, specific to preterm-born individuals, in cognitive-neurophysiological processes.

We have available new data from term-born siblings of the same preterm-born adolescents whom we previously compared to unrelated controls (James *et al*., 2017; Rommel *et al*., 2017, 2019). By comparing the preterm-born adolescents to their term-born siblings, we now aim to test the within-sibling associations of preterm birth with increased ADHD symptoms and the specific cognitive-neurophysiological impairments, to investigate if the relationships are independent of unmeasured familial factors shared by siblings.

## Method

### Sample

The preterm group was recruited from secondary schools in South East England, and was mainly Caucasian (84.6%) (Rommel *et al*., 2017). Preterm participants had one full sibling available for ascertainment and were born before 37 weeks' gestation. Exclusion criteria for all groups were IQ < 70, cerebral palsy or any other medical conditions that affect motor co-ordination, as well as brain disorders and any genetic or medical disorder that might mimic ADHD. Seven individuals from the preterm sample were excluded because medical birth records could not corroborate preterm status (GA ⩾ 37 weeks). One individual was excluded due to IQ < 70. The total eligible sample consisted of 145 sibling pairs (*n* = 290 participants).

For descriptive purposes, in line with previous studies (Cheung *et al*., 2016; Rommel *et al*., 2017), a research diagnosis of ADHD was made if participants scored six or more on either the inattention or hyperactivity-impulsivity parent-rated subscales of The Diagnostic Interview for ADHD in Adults (Kooij and Francken, 2007), and if they received two or more positive scores on two or more areas of impairment on the parent-rated Barkley Functional Impairment Scale (Barkley and Murphy, 2006). Seventeen preterm-born participants and seven term-born siblings met our criteria for a research ADHD diagnosis. Two of the preterm-born participants were taking ADHD medication, but a 48-h ADHD medication-free period was required prior to assessments.

The present analysis only uses siblings discordant for preterm birth. Therefore, the final sample in this analysis consisted of 208 participants: 104 preterm-born participants (GA ranged from 23 weeks to 36) and their 104 term-born siblings (GA ranged from 37 to 42 weeks). On average, the siblings in a pair were discordant by 5.97 (s.d. = 3.00) weeks of gestation. Of the sibling pairs, 45% (*n* = 47) were same-sex siblings. In total, 55% of the preterm-born siblings were older than their term-born sibling. A 48-h ADHD medication-free period was required prior to assessments. Written informed consent was obtained following procedures approved by the National Research Ethics Service Committee London – Bromley (13/LO/0068).

### Procedure

Participants attended a single 4.5 h research session, which included an electroencephalogram (EEG) assessment with cognitive tests and clinical interviews. As part of the EEG assessment, participants completed a cued continuous performance test (CPT) with flankers (CPT-OX) (Doehnert *et al*., 2010), an arrow flanker task with congruent (low-conflict) and incongruent (high-conflict) conditions (Albrecht *et al*., 2008; McLoughlin *et al*., 2009, 2014) and a four-choice reaction time task with two conditions, the fast task (Kuntsi *et al*., 2005; Andreou *et al*., 2007). Face-to-face clinical interviews were administered to the parent of each participant shortly before or after the participant's assessment.

### Measures

#### Gestational age

GA was obtained from Personal Child Health Records (also known in the UK as the ‘red book’), the national standard development record given to parents. Preterm birth was considered as <37 gestational weeks and term birth was considered ⩾37 weeks.

#### IQ and digit span

The vocabulary and block design subtests of the Wechsler Abbreviated Scale of Intelligence (WASI) (Wechsler, 1999) were administered to all participants to derive estimates of IQ. The digit span subtest from the WISC-III was administered to obtain digit span forward (DSF) and backwards (DSB) (Wechsler, 1991). DSB was used in this analysis and required the participant to verbally repeat a sequence of digits in the backwards order, and is a measure of verbal working memory.

#### ADHD symptoms

Parents were asked to rate the behaviour of each sibling using the Revised Conners' Parent Rating Scale (CPRS-R) (Conners *et al*., 1998). The CPRS-R has two DSM-IV symptom sub-scales (inattentiveness and hyperactivity-impulsivity), each consisting of nine items that map onto DSM-IV criteria. The sum of all 18 items calculates a total DSM-IV ADHD symptom score with a greater score indicating more ADHD symptoms.

#### Cued continuous performance test (CPT-OX)

The CPT-OX is a cued Go/NoGo task that probes attention, preparation and response inhibition (Doehnert *et al*., 2010). The task consisted of 400 black letter arrays, made up of a centre letter and incompatible flankers on each side to increase difficulty. Cue and target letters (‘O’ and ‘X’ respectively) were flanked by incompatible letters (‘XOX’ and ‘OXO’ respectively). Participants were instructed to ignore the flanking letters and respond as quickly as possible to only cue-target sequences (‘O’-‘X’). Eighty cues (‘XOX’) were followed by the target (‘OXO’) in 40 trials (Go condition), and by neutral distractors in the remainder of trials (NoGo condition).

#### The arrow flanker task

The task was an adaptation of the Eriksen flanker paradigm designed to increase cognitive load as used in previous studies (Albrecht *et al*., 2008; McLoughlin *et al*., 2009, 2014). In each trial, a central black fixation mark was replaced by a target arrow (a black 18 mm equilateral triangle). Participants had to indicate whether this arrow pointed towards the left or right by pressing corresponding response buttons with their left or right index fingers. Both flankers either pointed in the same (congruent) or opposite (incongruent) direction to the target. As such, conflict monitoring is maximal during the incongruent condition.

#### The fast task

Participants performed a four-choice RT task with a baseline condition (72 trials) with four empty circles (warning signals, arranged horizontally) first appearing for 8000 ms, after which one of them (the target) was coloured in (Andreou *et al*., 2007). Participants were asked to press the response key that directly corresponded to the position of the target. Following a response, the stimuli disappeared from the screen and a fixed inter-trial interval of 2.5 s followed. A comparison condition with a fast event rate (1 s) and incentives followed the baseline condition (Andreou *et al*., 2007). Cognitive-performance measures of MRT, and RTV (s.d. of RTs) were calculated for each condition.

#### EEG recording

The EEG was recorded from 62 channels DC-coupled recording system (extended 10–20 montage), with a 500 Hz sampling rate, impedances kept under 10 kΩ, and FCz as the recording reference electrode. The electrooculograms were recorded from electrodes above and below the left eye and at the outer canthi. The EEG data were analysed using a Brain Vision Analyzer (2.0) (Brain Products, Germany). After down-sampling the data to 256 Hz, the EEG data were re-referenced to the average (turning FCz into an active electrode) and filtered offline with digital band-pass (0.1 to 30 Hz, 24 dB/oct) Butterworth filters. Ocular artefacts were identified and removed from the data using Independent Component Analysis [ICA (Jung *et al*., 2000)]. All ERP averages contained at least 20 accepted sweeps. ERPs were extracted from the CPT-OX (CNV, Go-P3, NoGo-P3), flanker task (N2, Pe, ERN, incongruent condition only) and fast task (CNV amplitude and P3 amplitude) following procedures used on previous analyses of this sample (James *et al*., 2017; Rommel *et al*., 2017, 2019); see online Supplementary Material I for further details.

### Statistical analyses

ADHD symptoms and cognitive performance and event-related potential (ERP) measures which we have previously shown to be impaired in the preterm group (James *et al*., 2017; Rommel *et al*., 2017, 2019), compared to unrelated term-born controls, are included in this analysis (partly replicated in Table 2; see online Supplementary Material V for details about the unrelated term-born control group). We additionally investigated within-sibling associations for ADHD symptom sub-scales (inattentiveness and hyperactivity-impulsivity).

In order to reduce the number of statistical comparisons, in this analysis we only chose one electrode where the previous preterm-control differences were maximal (instead of electrodes at multiple sites), and consequently analysed Go-P3 from Pz only, CNV at CPz only in the CPT-OX, and N2 at Fz only. In addition, in the fast task, cognitive performance measures of MRT and RTV from the baseline condition only are included in this analysis, as the baseline condition is more sensitive to preterm-control differences in cognitive performance (James *et al*., 2017). Similarly, change measures in P3 and CNV from the fast task, from a baseline (slow, unrewarded) condition to a faster condition with rewards, were also omitted due to their high correlation with P3 and CNV in the fast-incentive condition. According to these criteria the following measures were retained for inclusion: IQ, verbal working memory (DSB); response preparation (CNV at CPz), executive response control (Go-P3 at Pz), response inhibition (NoGo-P3 at Cz) from the CPT-OX; conflict monitoring (N2 at Fz), conscious error processing (Pe at CPz), automatic error processing (ERN at FCz), from the flanker task; MRT and RTV (baseline condition), response preparation (CNV at CPz in the fast-incentive condition) and attention allocation (P3 at Pz in the fast-incentive condition) from the fast task.

The relationship of preterm birth with cognitive and ERP measures was investigated using a within-sibling fixed-effect design (Neuhaus and McCulloch, 2006; Lahey and D'Onofrio, 2010; Donovan and Susser, 2011; D'Onofrio *et al*., 2013), which models within-sibling pair differences in cognitive and neurophysiological measures as a function of within-pair differences of preterm birth, allowing the effect of preterm birth on cognitive and neurophysiological measures to be estimated while accounting for unmeasured confounding factors (i.e. all genetic and environmental factors that make siblings alike). Preterm birth was first studied as a dichotomous variable (preterm birth: born before <37 weeks gestation). See Table 1 for the overall sample mean and standard deviations (s.d.), and see online Supplementary Material II for group-level (term and preterm) means, but note this is not a group analysis. Models were fitted to standardised (*z*) cognitive and neurophysiological measures so that beta coefficients represent a standardised effect size measure. Therefore, one-unit change for the measure of preterm birth leads to a beta change in standard deviation in cognitive-neurophysiological measures. The effect size for the dichotomous analysis is comparable to Cohen's *d*. All analyses were conducted in Stata 13 software. Age and sex effects were regressed out from all analyses as is standard practice for quantitative family studies (Bouchard *et al*., 1990). IQ and birth order were used as additional covariates (online Supplementary Material III–IV).

**Table 1.** Descriptive statistics: means and standard deviations () for the overall sample

Variable	All *n* = 208	
Male (%)	141	(58%)
Age	15.07	(2.21)
Gestational age	35.57	(4.00)
ADHD symptoms (total)	7.34	(9.09)
IQ	104.72	(12.36)
DSB	6.43	(2.03)
Cued continuous performance test		
CNV (μV)	−2.68	(2.63)
Go-P3 (μV)	8.66	(4.62)
NoGo-P3 (μV)	8.10	(4.89)
Arrow flanker task		
N2 (μV)	−5.55	(3.65)
Pe (μV)	10.01	(5.56)
ERN (μV)	8.56	(4.57)
The fast task		
MRT (ms) in baseline	574.11	(155.82)
RTV (ms) in baseline	151.34	(135.14)
CNV (μV) in fast-incentive	−1.20	(1.71)
P3 (μV ms) in fast-incentive	1061.17	(920.07)

ADHD, attention-deficit/hyperactivity disorder; DSB, digit span backwards; MRT, mean reaction time in the baseline (slow, unrewarded) condition of the fast task; RTV, reaction time variability in the baseline (slow, unrewarded) condition of the fast task; CNV, contingent negative variation; Go-P3 = P3 amplitude; NoGo-P3 = P3 amplitude; N2 = N2 amplitude; Pe, positive related negativity in the incongruent condition; ERN, error-related negativity in the incongruent condition; CNV, contingent negative variation amplitude in the fast-incentive condition; P3 = P3 amplitude in the fast-incentive condition; ms, milliseconds, μV, microvolts.

### Role of the funding source

The funder of the study had no role in study design, data collection, data analysis, data interpretation or writing of the report. The corresponding author confirms that she has full access to all the data in the study and had final responsibility for the decision to submit for publication.

## Results

The within-sibling comparisons, which compare the preterm group to their term-born siblings, are new and the focus here but, for ease of comparison and completeness, we also report the statistics from comparisons between the preterm group and an unrelated control group (Table 2) [partly previously reported in James *et al*. (2017) and Rommel *et al*. (2017, 2019)].

**Table 2.** Standardised regression coefficients of the association between preterm birth, ADHD symptoms and standardised cognitive-event-related potential measures (controlling for age and sex). Preterm-probands are compared to (A) term-born siblings in a within-sibling fixed effect model and (B) unrelated controls in a linear regression model [originally partly reported in James *et al*. (2017) and Rommel *et al*. (2017, 2019)].

	(A) Within-sibling comparison (*n* = 208)			(B) Preterm v. unrelated control comparison (*n* = 321)		
Variable	*β*	*p*	95% CI	*β*	*p*	95% CI
ADHD symptoms (total)	0.32	**0.01**	0.05 to 0.58	0.64	**0.04**	0.17 to 2.05
IQ	−0.20	**0.04**	−0.40 to −0.01	−0.35	**0.02**	−0.64 to −0.06
DSB	−0.06	0.64	−0.30 to 0.18	−0.56	**<0.01**	−0.85 to −0.28
Cued continuous performance test						
CNV (μV)	0.71	**0.02**	0.14 to 1.18	0.87	**<0.01**	0.42 to 1.33
Go-P3 (μV)	−0.34	**0.01**	−0.61 to −0.10	−0.42	**<0.01**	−0.68 to −0.16
NoGo-P3 (μV)	−0.07	0.45	−0.33 to 0.15	−0.58	**<0.01**	−0.86 to −0.30
Arrow flanker task						
N2 (μV)	0.30	**0.04**	0.02 to 0.54	0.79	**<0.01**	0.51 to 1.05
Pe (μV)	−0.35	**0.02**	−0.52 to −0.05	−0.67	**<0.01**	−0.95 to −0.39
ERN (μV)	−0.29	**0.03**	−0.03 to −0.55	−0.40	**<0.01**	−0.74 to −0.07
The fast task						
MRT (ms) in baseline	0.37	**0.03**	0.03 to 0.72	0.30	**<0.01**	0.15 to 0.47
RTV (ms) in baseline	0.45	**0.01**	0.09 to 0.80	0.35	**<0.01**	0.13 to 0.57
CNV (μV) in fast-incentive	0.22	**0.05**	0.00 to 0.43	0.92	**<0.01**	0.58 to 1.26
P3 (μV ms) in fast-incentive	−0.26	**0.05**	−0.53 to −0.01	−0.85	**<0.01**	−1.11 to −0.58

*Note*: *p* < 0.05 indicated in bold.

ADHD, attention-deficit/hyperactivity disorder; DSB, digit span backwards; MRT, mean reaction time in the baseline (slow, unrewarded) condition of the fast task; RTV, reaction time variability in the baseline (slow, unrewarded) condition of the fast task; CNV, contingent negative variation; Go-P3 = P3 amplitude; NoGo-P3 = P3 amplitude; N2 = N2 amplitude; Pe, positive related negativity in the incongruent condition; ERN, error-related negativity in the incongruent condition; CNV, contingent negative variation amplitude in the fast-incentive condition; P3 = P3 amplitude in the fast-incentive condition; ms, milliseconds, μV, microvolts.

Models were fitted to standardised (*z*) cognitive and neurophysiological measures so that beta coefficients presented represent a standardised effect size measure.

Within-sibling comparisons showed that those who were born preterm were more likely to have increased parent-rated total ADHD symptoms [and on both inattentiveness (*β* = 0.34, 95% CI 0.07 to 0.60) and hyperactivity-impulsivity (*β* = 0.24, 95% CI 0.04 to 0.51) ADHD symptom sub-scales]; lower IQ, as well as decreased CNV amplitude and decreased Go-P3 amplitude on the CPT-OX task, decreased N2 amplitude and decreased Pe and ERN on the flanker task, increased MRT and RTV in the baseline condition of fast task, and decreased CNV amplitude and P3 amplitude on the fast-incentive condition of fast task, independent of familial factors (Table 2). Unlike the unrelated-comparisons, within-sibling comparisons demonstrated that those who were born preterm were not more likely to be impaired on DSB (*β* = −0.05, 95% CI −0.30 to 0.18) or NoGo-P3 amplitude (*β* = −0.07, 95% CI −0.33 to 0.15) on the CPT-OX, compared to their term-born siblings; these within-sibling associations were therefore largely attenuated compared to the unrelated comparisons (Table 2). Post-hoc comparisons between term-born siblings of the preterm-probands and unrelated controls further demonstrated that, after adjusting for sex and age, the term-born siblings had lower DSB (*β* = −0.48, 95% CI −0.74 to −0.21) and NoGo-P3 amplitude (*β* = −0.55, 95% CI −0.83 to −0.26), compared to the unrelated control group; the results remained similar when IQ was additionally adjusted (*β* = −0.56, 95% CI −0.85 to −0.28 and *β* = −0.53, 95% CI −0.83 to −0.24, respectively).

The pattern of results remained similar when IQ and birth order were included as additional covariates (online Supplementary Material III–IV).

## Discussion

In this sibling-comparison study of adolescents, we find evidence for significant associations of preterm birth with increased ADHD symptoms, including both inattentiveness and hyperactivity-impulsivity sub-scales, and with specific cognitive-neurophysiological impairments, including IQ, preparation-vigilance processes (RTV, MRT, CNV) and error and conflict processing (N2, Pe, ERN), which are independent of familial factors shared by siblings, indicating that preterm birth is likely in the causal pathway leading to these identified impairments. In contrast, the association of preterm birth with executive control measures of working memory (DSB) and inhibition (NoGo-P3) was not independent of familial factors, indicating that these previously obtained associations (James *et al*., 2017; Rommel *et al*., 2017, 2019) are more likely to be due to other characteristics that differentiate families with a preterm-born child from other families, rather than preterm birth *per se*.

The robust association between preterm birth and increased ADHD symptoms, when controlling for unmeasured familial confounding factors, is in line with a previous finding using this sibling-comparison approach (D'Onofrio *et al*., 2013). Whilst multiple studies have indicated that preterm birth is associated with decreased IQ (Kerr-Wilson *et al*., 2012), we show that this relationship is independent of potential shared familial confounds such as socio-economic status (Goldenberg *et al*., 2008), consistent with a causal inference. We also obtained evidence of independent associations of preterm birth with cognitive and neurophysiological measures indexing preparation vigilance processes (MRT, RTV, CNV), response execution and attention allocation (Go-P3, P3), conflict monitoring (N2) and error processing (Pe, ERN), when controlling for unmeasured familial confounding factors. Future research should explore the specific mechanisms whereby preterm birth leads to these cognitive-neurophysiological impairments and increased ADHD symptoms. For example, as brain connections strengthen throughout the third trimester (29 to 40 weeks gestation) (Ball *et al*., 2014; van den Heuvel *et al*., 2014), giving birth prematurely could feasibly result in disruption of developing brain networks associated with ADHD, as well as disruption of other networks associated with additional impairments. In order to help identify and provide support for those at risk, our findings support the notion that there should be greater awareness amongst medical and educational professionals about the increased risk of cognitive-neurophysiological impairments in people born preterm (Henderson *et al*., 2012; Brogan *et al*., 2014; Johnson *et al*., 2014). It is striking that even within our sample, who were a relatively well-functioning sample recruited from mainstream schools, impairments are still observed in preterm-born individuals at least a decade after the preterm birth event.

In contrast, we showed no significant associations between preterm birth and decreased verbal working memory (DSB) and attenuated response inhibition (NoGo-P3) when controlling for unmeasured familial confounding factors. This suggests that familial factors shared by siblings, which include factors correlated with preterm birth (i.e. maternal genetic risk for giving birth preterm, socio-economic status, family upbringing, and other genetic and environmental factors that are shared by members of the same family), may account for these deviations with preterm birth previously observed in preterm individuals when compared to unrelated controls. The finding that working memory is not on the causal pathway from preterm birth is in line with results from a study which demonstrated that a combined measure of short-term auditory memory and verbal working memory (combining DSF and DSB) were not on the causal pathway between birth weight and ADHD symptoms in adolescents (Morgan *et al*., 2016). Future research should aim to identify the background risk factors that characterise families with a preterm-born child and account for the impairments that distinguish them from families without preterm-born children.

The present study is, to our knowledge, the first study investigating the effects of preterm birth on cognitive-neurophysiological measures in adolescents in a quasi-experimental sibling design, which is essential for drawing stronger causal inferences. However, as is the case for all such non-randomised quasi-experimental studies, we cannot rule out all confounding factors (D'Onofrio *et al*., 2013). Whilst we control for shared maternal risk factors for preterm birth, we were unable to investigate whether specific maternal risk factors (e.g. trauma, smoking) differed between pregnancies. As siblings only share ~50% of DNA, the sibling-comparison design does not control for sibling-specific genetic influences that could influence preterm birth and, as such, the causal role of genetic influences cannot be ruled out. However, studies have suggested a negligible role of the foetus' genotype in determining GA (Svensson *et al*., 2009). Further, whilst our adolescent sample offers a unique perspective, it would be informative to have follow-up assessments when all participants have reached adulthood.

In conclusion, our findings provide novel insight into the potential causal pathways to cognitive-neurophysiological impairments and increased ADHD symptoms in adolescents born preterm. By distinguishing impairments that are consistent with a causal inference of preterm birth from those that are instead linked to background characteristics of families with a preterm-born child, these results suggest that interventions need to target both preterm-birth specific and family-level risk factors to minimise impairments in those at risk. If replicated, clinical implications include raising the awareness about the increased risk of cognitive and brain impairments in preterm-born children; and understanding the increased risk of IQ and cognitive/brain impairments are likely causally related to preterm birth itself, whereas executive function impairments are via different causal mechanisms associated with families at risk.

## Supplementary material

For supplementary material accompanying this paper visit https://doi.org/10.1017/S0033291719001211. click here to view supplementary material
