# Supplementary material for: Is association of preterm birth with cognitive-neurophysiological impairments and ADHD symptoms consistent with a causal inference or due to familial confounds?
Source: Psychol Med. 2019 Jun 3;50(8):1278–84. doi: 10.1017/S0033291719001211 (PMC7322548; doi:10.1017/S0033291719001211)
Supplement: Supplementary file 1 [file S0033291719001211sup001.docx]

**SUPPLEMENTARY MATERIAL CONTENT**

# SUPPLEMENTARY MATERIAL I: Event related potential (ERP) and skin conductance (SC) extraction.

***Cued continuous performance test (CPT-OX)***

For the CPT-OX task, stimulus-locked epochs (stimulus window from −200 to 1650ms) were averaged based on three different response conditions: Cue, Go and NoGo. Averages were calculated for trials with correct responses (Go) or correctly rejected trials (NoGo and Cue), which included at least 20 artefact-free segments. Based on previous research (McLoughlin et al. 2010; Doehnert et al. 2013; Albrecht et al. 2013), ERP measures were identified within selected electrodes and latency windows for which effects were expected to be largest. These measures were then confirmed separately for the three groups using topographic maps. In Cue trials, the P3 was measured at Pz between 300-650ms, and the CNV was measured at Cz and CPz between 1300-1650ms. In Go trials, the P3 was measured at CPz and Pz between 250-500ms. No clear N2 was observed in Go trials, consistent with other studies employing tasks with low conflict-monitoring demands, and was, therefore, not included in the analysis. In NoGo trials, the P3 was measured at FCz and Cz between 250-550ms and the N2 was measured at Fz between 175-325ms. The CNVs were analysed as mean amplitudes between 1300 and 1650ms following cues over the central electrode (CPz). The Cue-P3 had a parietal maximum and was defined as the most positive peak between 250 and 600 ms following cue trials at electrode Pz. The NoGo-P3 was defined as the most positive peak between 250 and 600 ms following NoGo trials at electrode Cz.

***The (arrow) flanker task***

Analyses of ERPs of performance monitoring in the flanker task were restricted to the incongruent condition, as the task used in this study is known to elicit strong N2, error related negativity (ERN) and positivity (Pe) components in high-conflict, but not in low-conflict, conditions. Baseline correction was applied using the -300 to -100 ms pre-target (-200 to 0 ms pre-flanker) interval, following the protocol of previous ERP analyses on the flanker task. Data were segmented based on (1) stimulus-locked incongruent trials where a correct response was made and (2) response-locked (error-related) incongruent trials where an incorrect response was made. Individual averages were created based on each condition, requiring ≥ 20 clean segments for each participant. After averaging, the electrodes and latency windows for ERP analyses were selected based on previous studies (Albrecht et al. 2008; McLoughlin et al. 2009; Groom et al. 2010; Nieuwenhuis et al. 2001, Michelini et al. 2016) topographic maps and the grand averages. The N2 was measured as maximum negative peak at Fz and FCz between 250-450 ms after target onset. The ERN was defined with respect to the preceding positivity (PNe, -100-50 ms) in order to obtain a more robust measure of this component, and was measured at FCz between 0-150 ms. The Pe was measured as maximum positive peak at CPz between 150-450 ms after an erroneous response on incongruent trials.

***The fast task (Andreou et al. 2007, Kuntsi et al 2005)***

In the fast task, P3 amplitude was analysed as the area amplitude measure (μV*ms) at Pz between 250 and 450ms, to reduce bias due to the varying noise levels induced by the different task conditions (Luck, 2005). For the P3 analyses, all the accepted trials were baseline-corrected using a pre-stimulus baseline of 200ms. The mean amplitudes of this pre-target period (-200ms - 0ms, using a technical zero baseline as in previous CNV work (7,8)) at Cz were also analysed separately as a CNV measure. This short interval not only corresponded to the P3 baseline, but also captured the short CNV in the fast-incentive condition with its one-second cue – target interval (Cheung et al. 2017; James, Cheung, Rijsdijk, et al. 2016).

# SUPPLEMENTARY MATERIAL II:

# TABLE 1: Descriptive statistics: means and standard deviations () for the preterm and term groups. NB this is not a group analysis.

| **Variable** | | **Term**  **n=104** | | **Preterm**  **n=104** | |
| --- | --- | --- | --- | --- | --- |
|  | Male (%) | 60 | (58%) | 59 | (57%) |
|  | Age | 15.02 | (2.44) | 15.10 | (2.03) |
|  | Gestational age | 39.19 | (1.22) | 32.88 | (3.14) |
|  | ADHD symptoms (total) | 6.97 | (9.23) | 7.45 | (9.02) |
|  | ADHD symptoms (inattentive) | 4.53 | (6.01) | 4.85 | (6.01) |
|  | ADHD symptoms (hyperactivity-impulsivity) | 2.44 | (3.92) | 2.64 | (3.91) |
|  | IQ | 105.86 | (11.33) | 103.86 | (13.06) |
|  | DSB | 6.45 | (1.91) | 6.30 | (2.14) |
| **Cued continuous performance test** | |  |  |  |  |
|  | CNV (µV) | -8.06 | (3.79) | 7.38 | (3.48) |
|  | Go-P3 (µV) | 9.22 | (4.24) | 8.21 | (4.88) |
|  | NoGo-P3 (µV) | 8.17 | (4.95) | 8.04 | (4.86) |
| **Arrow flanker task** | |  |  |  |  |
|  | N2 (µV) | -5.01 | (4.02) | -4.34 | (4.22) |
|  | Pe (µV) | 8.47 | (4.56) | 8.71 | (4.67) |
|  | ERN (µV) | 9.05 | (3.40) | 8.71 | (4.12) |
| **The fast task** | |  |  |  |  |
|  | MRT (ms) in baseline | 551.05 | (132.40) | 590.45 | (169.12) |
|  | RTV (ms) in baseline | 134.25 | (99.97) | 163.43 | (58.38) |
|  | CNV (µV) in fast-incentive | -1.13 | (1.70) | -1.04 | (1.77) |
|  | P3 (µV*ms) in fast-incentive | 1061.17 | (920.07) | 1018.06 | (872.97) |

# SUPPLEMENTARY MATERIAL III:

# Within-siblings, fixed effect model of preterm birth on standardised scores (controlling for age, sex and IQ) (n = 208).

|  | Variable | β Coef | P | 95% CI |
| --- | --- | --- | --- | --- |
|  | ADHD symptoms | **0.24** | **0.02** | **0.03,0.49** |
|  | DSB | -0.04 | 0.67 | -0.17,0.27 |
|  |  |  |  |  |
| **Cued continuous performance test** | |  |  |  |
|  | CNV (µV) | **0.59** | **0.03** | **0.06, 1.12** |
|  | Go-P3 (µV) | **-0.32** | **0.02** | **-0.58,-0.06** |
|  | NoGo-P3 (µV) | -0.02 | 0.86 | -0.26,-0.22 |
|  |  |  |  |  |
| **Arrow flanker task** | |  |  |  |
|  | N2 (µV) | **0.27** | **0.04** | **0.01,0.54** |
|  | Pe (µV) | **-0.23** | **0.05** | **-0.46,-0.00** |
|  | ERN (µV) | **0.28** | **0.04** | **0.02,-0.54** |
|  |  |  |  |  |
| **The fast task** |  |  |  |  |
|  | MRT (ms) in baseline | **0.26** | **0.05** | **0.02,0.53** |
|  | RTV (ms) in baseline | **0.32** | **0.04** | **0.02,0.67** |
|  | CNV (µV) in fast-incentive | **0.41** | **0.01** | **0.22,0.61** |
|  | P3 (µV*ms) in fast-incentive | **-0.24** | **0.04** | **-0.48, -0.01** |

Note: p<0.05 indicated in bold. ADHD=attention-deficit/hyperactivity disorder; DSB=digit span backwards; MRT=mean reaction time in the baseline (slow, unrewarded) condition of the fast task; RTV=reaction time variability in the baseline (slow, unrewarded) condition of the fast task; CNV=contingent negative variation; Go-P3=P3 amplitude; NoGo-P3=P3 amplitude; N2=N2 amplitude; Pe=positive related negativity in the incongruent condition; ERN=error related negativity in the incongruent condition; CNV=contingent negative variation amplitude in the fast-incentive condition; P3=P3 amplitude in the fast-incentive condition; ms=milliseconds, µV=microvolts. Models were fitted to standardised (z) cognitive and neurophysiological measures so that beta coefficients presented represent a standardized effect size measure.

# SUPPLEMENTARY MATERIAL III:

# Within-siblings, fixed effect model of preterm birth on standardised scores (controlling for age, sex, IQ and birth order) (n = 208).

|  | Variable | β Coef | P | 95% CI |
| --- | --- | --- | --- | --- |
|  | ADHD symptoms | **0.23** | **0.02** | **0.03,0.50** |
|  | DSB | 0.01 | 0.98 | -0.21,0.29 |
|  |  |  |  |  |
| **Cued continuous performance test** | | |  |  |
|  | CNV (µV) | **0.59** | **0.03** | **0.06, 1.12** |
|  | Go-P3 (µV) | **-0.32** | **0.02** | **-0.58,-0.06** |
|  | NoGo-P3 (µV) | -0.02 | 0.86 | -0.26,-0.22 |
|  |  |  |  |  |
| **Arrow flanker task** | |  |  |  |
|  | N2 (µV) | **0.27** | **0.04** | **0.01,0.54** |
|  | Pe (µV) | **-0.23** | **0.05** | **-0.46,-0.00** |
|  | ERN (µV) | **0.28** | **0.04** | **0.02,-0.54** |
|  |  |  |  |  |
| **The fast task** | |  |  |  |
|  | MRT (ms) in baseline | **0.26** | **0.05** | **0.02,0.53** |
|  | RTV (ms) in baseline | **0.32** | **0.04** | **0.02,0.67** |
|  | CNV (µV) in fast-incentive | **0.41** | **0.01** | **0.22,0.61** |
|  | P3 (µV*ms) in fast-incentive | **-0.24** | **0.04** | **-0.48, -0.01** |

Note: p<0.05 indicated in bold. ADHD=attention-deficit/hyperactivity disorder; DSB=digit span backwards; MRT=mean reaction time in the baseline (slow, unrewarded) condition of the fast task; RTV=reaction time variability in the baseline (slow, unrewarded) condition of the fast task; CNV=contingent negative variation; Go-P3=P3 amplitude; NoGo-P3=P3 amplitude; N2=N2 amplitude; Pe=positive related negativity in the incongruent condition; ERN=error related negativity in the incongruent condition; CNV=contingent negative variation amplitude in the fast-incentive condition; P3=P3 amplitude in the fast-incentive condition; ms=milliseconds, µV=microvolts. Models were fitted to standardised (z) cognitive and neurophysiological measures so that beta coefficients presented represent a standardized effect size measure.

# SUPPLEMENTARY MATERIAL IV

***Control sample description (Replicated from Rommel et al. 2016).***

ADHD and control sibling pairs, who had taken part in our previous research (11,12), were invited to take part in a follow-up study (Cheung et al. 2016). Exclusion criteria for all groups were IQ of <70, cerebral palsy or any other medical condition that affects motor coordination including epilepsy, as well as brain disorders and any genetic or medical disorder that might mimic ADHD. All participants were of white European descent and had one full sibling available for ascertainment. Here only those in the control group who were term-born are compared to a group of preterm-born adolescents. The control group was initially recruited from primary (aged 6–11 years) and secondary (aged 12–18 years) schools in the United Kingdom, aiming for an age and sex match with the ADHD sample. Control individuals and their siblings were included in the control group if they did not meet DSM-IV criteria for any ADHD subtype either in childhood or at follow-up.

# REFERENCES

1. Albrecht B, Brandeis D, Uebel H, et al. Action monitoring in boys with attention-deficit/hyperactivity disorder, their nonaffected siblings, and normal control subjects: evidence for an endophenotype. *Biol. Psychiatry*. 2008;64(7):615–25.

2. McLoughlin G, Albrecht B, Banaschewski T, et al. Performance monitoring is altered in adult ADHD: A familial event-related potential investigation. *Neuropsychologia*. 2009;47(14):3134–3142.

3. Groom MJ, Cahill JD, Bates AT, et al. Electrophysiological indices of abnormal error-processing in adolescents with attention deficit hyperactivity disorder (ADHD). *J. Child Psychol. Psychiatry.* 2010;51(1):66–76.

4. Nieuwenhuis S, Ridderinkhof KR, Blom J, et al. Error-related brain potentials are differentially related to awareness of response errors: evidence from an antisaccade task. *Psychophysiology*. 2001;38(5):752–60.

5. Michelini G, Kitsune GL, Cheung CHM, et al. ADHD Remission is Linked to Better Neurophysiological Error Detection and Attention-Vigilance Processes. *Biol. Psychiatry*. 2016;

6. Andreou P, Neale BM, Chen W, et al. Reaction time performance in ADHD: improvement under fast-incentive condition and familial effects. *Psychol. Med.* 2007;37(12):1703–15.

7. Albrecht B, Brandeis D, Uebel H, et al. Familiality of neural preparation and response control in childhood attention deficit-hyperactivity disorder. *Psychol. Med.* 2013;43(9):1997–2011.

8. Banaschewski T, Brandeis D, Heinrich H, et al. Association of ADHD and conduct disorder--brain electrical evidence for the existence of a distinct subtype. *J. Child Psychol. Psychiatry.* 2003;44(3):356–76.

9. Cheung CHM, McLoughlin G, Brandeis D, et al. Neurophysiological Correlates of Attentional Fluctuation in Attention-Deficit/Hyperactivity Disorder. *Brain Topogr.* 2017;

10. James S-N, Cheung CHM, Rijsdijk F, et al. Modifiable Arousal in ADHD and its Etiological Association with Fluctuating Reaction Times. *Biol. Psychiatry Cogn. Neurosci. Neuroimaging*. 2016;

11. Chen W, Zhou K, Sham P, et al. DSM-IV combined type ADHD shows familial association with sibling trait scores: a sampling strategy for QTL linkage. *Am. J. Med. Genet. B. Neuropsychiatr. Genet.* 2008;147B(8):1450–60.

12. Kuntsi J, Wood AC, Rijsdijk F, et al. Separation of cognitive impairments in attention-deficit/hyperactivity disorder into 2 familial factors. *Arch. Gen. Psychiatry*. 2010;67(11):1159–67.
